# Hypnosis and Virtual Reality in the treatment of a MSNA (unaccompanied foreign minor)

**DOI:** 10.1192/j.eurpsy.2025.1659

**Published:** 2025-08-26

**Authors:** S. Bellissima, G. Pellegrino

**Affiliations:** 1 Medicina della Migrazione e delle Emergenze Sanitarie, ASP 3 Catania, Catania, Italy

## Abstract

**Introduction:**

11 years old, Syrian, he arrives alone in Italy after disembarking in Lampedusa, coming from Libya where he reports having stayed for a year. He is assigned to a community by the Juvenile Court of Catania.

A few days later, he exhibits multiple episodes of chest pain, difficulty breathing, drooling, muscle rigidity and tremors, and in the most severe case, he is taken to the emergency room and then hospitalized in pediatric neuropsychiatry.

Diagnosis ICD 10: Dissociative disorders (Code F44); Post-traumatic stress disorder (Code F53.1). Trazodone hydrochloride (60 ml) is prescribed, 5 drops three times a day.

**Objectives:**

Care of the person and resolution of symptoms through psychotherapeutic management by the “Medicina delle Migrazione e delle Emergenze Sanitarie” of ASP 3 Catania (Italy).

**Methods:**

Hypnosis combined with virtual reality, a technique already experimented with by the writer, the procedure consists of: trance induction; awakening; application of the visor; re-induction of the trance through conditioning, simultaneously with the departure of the virtual stimulus; continuous feedback with peripheral device. The stimulus situations transmitted by the viewer have as object setting such as: Abstract: lights, colors, geometric shapes; Concrete: naturalistic and aquatic landscapes, animals, guided tours, etc.

**Results:**

He is a good hypnotic subject, responds well and after initial disorientation, benefits from psychotherapy, showing a slow but continuous improvement in behavior: anxiety progressively decreases, oppositional and rebellious behavior in the community wanes, conduct at school becomes appropriate where there were previously conflicts, sleep changes from disturbed to regular, and he interacts positively with adults and peers.

Marked interference is noted via phone from the family of origin, urging him to go to Germany where a half-sister resides (the procedure is feasible but very complex), leading to a resurgence of symptoms and feelings of guilt in the minor, who perceives himself as unable to meet the demands of his relatives.

During Ramadan (he is Muslim), there are difficulties with concentration and lack of energy due to the lack of food and water, leading to a temporary delay in the work.

In an advanced phase of treatment, the minor categorically refuses to come to the clinic, probably due to being mocked by peers in the community, and it is decided to discontinue the sessions, especially since the work had progressed effectively and there was already an intention to gradually discontinue it.

**Image 1:**

**Figure:**
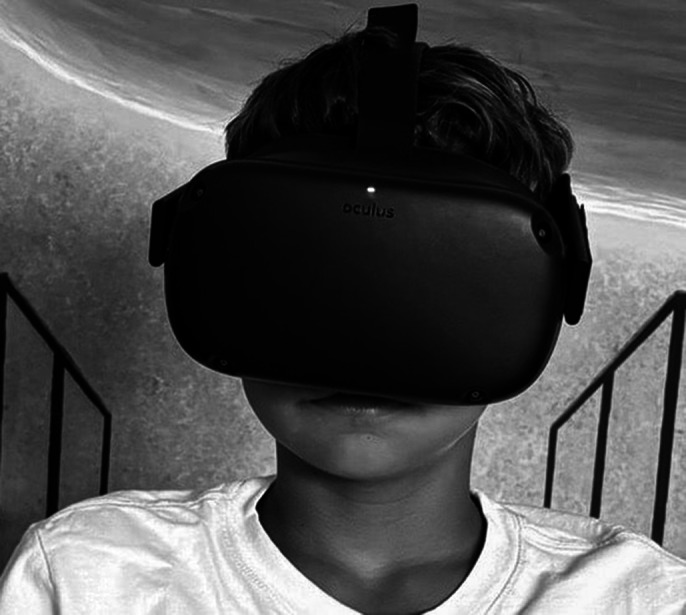

**Conclusions:**

It is believed that the use of hypnosis combined with virtual reality represents an appropriate treatment for post-traumatic stress disorder (PTSD), as it reduces anxiety, strengthens the ego, accelerates the process of change, and directs life in a positive direction.

The Juvenile Court has granted family reunification in Germany, and at present, the minor is awaiting departure.

**Disclosure of Interest:**

None Declared

